# Supplementary material for: Potential application of Aloe Vera-derived plant-based cell in powering wireless device for remote sensor activation
Source: PLoS One. 2019 Dec 27;14(12):e0227153. doi: 10.1371/journal.pone.0227153 (PMC6934303; doi:10.1371/journal.pone.0227153)
Supplement: Attachment — Submitted filename: Plos One_PONE-D-19-24896_v2.docx [file pone.0227153.s002.docx]

**Reviewer Comments**

**Manuscript Reference No.:** **PONE-D-19-24896**

**Title:** Potential application of Aloe Vera-derived plant-based cell in powering wireless device for remote sensor application

The manuscript presents a study on the design of a power management circuit from *Aloe barbadensis Miller* plant to remotely power a temperature and humidity sensor using wireless sensor network. In this manuscript the authors evaluated materials and methods to harvest energy optimally from *Aloe barbadensis Miller* leaves using Cu-Zn electrode pairs. Furthermore, the authors studied on the power management design to remotely power up the sensor with the energy derived from *Aloe barbadensis Miller* leaves. Overall, this paper outlines the voltage, current and power output based on the optimum setup in terms of number of Cu-Zn electrode pairs and extend to study its performance in a power management circuit (i.e. duty cycle, efficiency) based on power gating technique. The authors have answered the comments outlined though there are quite a number of grammatically errors found within the manuscript. In conclusion, the reviewer is satisfied with the revision of this manuscript and recommends the manuscript undergo minor revision to address the grammatical errors before it can be published on PLoS One. Below are few of the examples for the authors’ reference:

1. Pg. 3: "The boost converter that was built on using a 130 nm CMOS process could boost the voltage harvested..."
2. Pg. 3: "It is shown that the system could power the circuit in a nanoscopic scale due to is minute energy."
3. Pg. 4 and 5: "...harvest electrical energy measured to be higher than 3 V and 1 mA from the Aloe Vera plant..."
4. Page 11: “This is due to each of the leaves which behave as a cell contains some internal resistance.”
5. Page 11: “When more leaves are connected in parallel, the total internal resistance reduces and the admittance increases, thus allowing the increment of current as defined in Ohms law which state that when resistance decreases, current will increase.”
