# Supplementary material for: Potential application of Aloe Vera-derived plant-based cell in powering wireless device for remote sensor activation
Source: PLoS One. 2019 Dec 27;14(12):e0227153. doi: 10.1371/journal.pone.0227153 (PMC6934303; doi:10.1371/journal.pone.0227153)
Supplement: Attachment — Submitted filename: 3. Response to Reviewers.docx [file pone.0227153.s003.docx]

**Rebuttal Letter: Response to Reviewers PLOS ONE**

Dear respected PLOS ONE Academic Editor and Reviewers;

Good day. Thank you for your kind comments which provide valuable insights to improve my current manuscript quality. Each of the comments given is well analyze and provided with necessary explanation, correction and response to support the points added into the manuscript. Hence, attach here is the comments and responses provided for your kind review and checking.

**Comments & Response for Reviewer 1:**

Reviewer #1: The author had carefully addressed to all of my comments. I recommend an acceptance to its current form.

**Response:** No correction needed.

**Comments & Response for Reviewer 1:**

Reviewer #2: The comments outlined have been addressed clearly. However, the reviewer recommends the manuscript to undergo some minor revisions to address the grammatical errors. Below are few of the examples for the authors’ reference:

1. Pg. 3: "The boost converter that was built on using a 130 nm CMOS process could boost the voltage harvested..."

**Response:** The sentence is corrected to be:

**“**The boost converter that was built via a 130 nm CMOS process could boost the voltage harvested…”

2. Pg. 3: "It is shown that the system could power the circuit in a nanoscopic scale due to is minute energy."

**Response:** The sentence is corrected to be:

“It was shown that the system could power the circuit in a nanoscale due to its minute energy.”

3. Pg. 4 and 5: "...harvest electrical energy measured to be higher than 3 V and 1 mA from the Aloe Vera plant..."

**Response:** The sentence is corrected to be:

“…harvest electricity which is higher than 3 V and 1 mA from the Aloe Vera plants…”

4. Page 11: “This is due to each of the leaves which behave as a cell contains some internal resistance.”

**Response:** The sentence is corrected to be:

“It is because each of the leaves which act as a cell contains some internal resistance.”

5. Page 11: “When more leaves are connected in parallel, the total internal resistance reduces and the admittance increases, thus allowing the increment of current as defined in Ohms law which state that when resistance decreases, current will increase.”

**Response:** The sentence is corrected to be:

“When more leaves are connected in parallel, the total internal resistance reduces and the admittance increases. This allows the increment of current as defined in Ohms law which states that when resistance decreases, the current will increase.”
